# Stress, Anxiety, and Depression as Psychological Distress Among College and Undergraduate Students: A Scoping Review of Reviews Guided by the Socio-Ecological Model

**DOI:** 10.3390/healthcare13161948

**Published:** 2025-08-09

**Authors:** Sharmistha Roy, Ashis Kumar Biswas, Manoj Sharma

**Affiliations:** 1 Department of Social and Behavioral Health, School of Public Health, University of Nevada, Las Vegas, NV 89119, USA; 2 Department of Epidemiology and Biostatistics, School of Public Health, University of Nevada, Las Vegas, NV 89119, USA; 3 Department of Internal Medicine, Kirk Kerkorian School of Medicine at UNLV, University of Nevada, Las Vegas, NV 89102, USA

**Keywords:** college students, stress, anxiety, depression, mental health, scoping review, socio-ecological model

## Abstract

**Background/Objectives**: College and undergraduate students around the world struggle with stress, anxiety, and depression, which have a significant negative influence on their academic performance, social interactions, and general well-being. Creating successful preventative and intervention plans requires an understanding of the many, multi-level factors that contribute to psychological discomfort. The objective of this scoping review was to use the Socio-Ecological Model (SEM) to map the determinants of psychological distress among college students in a comprehensive manner. **Methods**: A total of 15 review publications published between 2015 and 2024, including narrative reviews, systematic reviews, meta-analyses, and umbrella reviews, were analyzed under the guidance of PRISMA ScR. These studies synthesized evidence across various countries, including China, Iran, India, Canada, Egypt, Nigeria, Saudi Arabia, and the United States. **Results**: Academic pressure, financial stress, poor sleep, unhealthy coping mechanisms, and pre-existing mental health issues were all individual-level concerns, with female and minority students being more vulnerable. Strong familial ties and friendships served as protective interpersonal support. Heavy academic workloads, strict grading guidelines, a lack of mental health resources, and unwelcoming campus environments were among the institutional factors. Stigma and socioeconomic disparities are examples of community-level variables that make mental health issues worse. **Conclusions**: Student mental health is shaped by interrelated factors across all SEM levels. Integrated, multi-level strategies are essential to fostering supportive campuses, strengthening community networks, and implementing inclusive policies that promote mental health equity.

## 1. Introduction

Mental health among college students has become a significant public health concern in the 21st century [1]. Higher education students are increasingly reporting psychological distress symptoms, such as stress, anxiety, and depression. These symptoms significantly affect their relationships, academic performance, physical health, and future opportunities [1,2].

Recent data indicate that college students in the United States are showing worsening tendencies in their mental health. As reported by the Healthy Minds Study 2023–2024 Data Report [1], the percentage of people with positive mental health fell from 51% in 2013–14 to 38% in 2023–24. Suicide planning doubled to 6%, serious suicidal thoughts climbed to 13%, and anxiety and depression symptoms increased to 34% and 38%, respectively. The American College Health Association (2024) reported that 76% of students experienced moderate to high levels of stress in the spring of 2024, while 49% reported feeling lonely. Less than half (46%) received counseling, despite 47% screening positive for anxiety or depression [3,4].

In a 2024 report by the American College Health Association, nearly half of college students in the United States stated that they felt so depressed it was difficult to function, while over 60% experienced overwhelming anxiety [3]. Approximately 35.1% of students reported that anxiety significantly hindered their academic performance, and 34.6% had been diagnosed with an anxiety disorder [5]. A meta-analysis conducted by Chang et al. (2021) further found that 31.0% of college and university students in Europe, America, and the Asia–Pacific region suffer from anxiety [6].

However, a higher anxiety prevalence of 55.0% was reported by another systematic review and meta-analysis that focused on students in Europe [6]. These statistics echo a global trend, as universities in Asia, Europe, Africa, and Latin America have similarly reported rising rates of psychological distress among students [7,8]. During and after the COVID-19 pandemic, these concerning trends have gotten worse, raising concerns about the mental health system and higher education systems’ capacity to promote students’ well-being in a variety of contexts. The COVID-19 pandemic (2020–2022) was especially stressful for college students [8].

Social distancing mandates, abrupt transitions to online learning, financial uncertainty, and isolation from peers and family contributed to widespread mental health deterioration [7]. In many countries, students faced multiple and intersecting stressors, some of which are unique to their educational status, while others reflect broader social and economic conditions. These included limited access to counseling services, digital divide issues, job loss, and, for international students, visa and travel disruptions. As a result, there has been a proliferation of research and review articles on mental health in higher education, particularly focusing on psychological distress during the pandemic years [8]. However, it is challenging to derive thorough and valuable insights across a range of contexts because of the fragmented nature of these investigations.

Although there are many review articles discussing different aspects of psychological distress among college students, the majority only discuss select features of individual symptoms, demographic factors, or individual intervention approaches [5]. The larger social, institutional, and policy environments in which pupils live and learn are ignored in these assessments. Integrative frameworks are urgently needed to reflect the complexity of interacting variables, as mental health is a complex and contextually embedded phenomenon [8,9].

One such framework is the Socio-Ecological Model (SEM), which conceptualizes human behavior and health outcomes as the result of dynamic interplays across multiple layers of influence: individual, interpersonal, institutional (organizational), community, and policy. This model has been widely used in public health, education, and psychology to examine health disparities, guide multi-level interventions, and inform system-oriented policy development [10].

The SEM is well suited to understanding college student mental health because it considers how personal characteristics (e.g., coping skills, self-efficacy), social relationships (e.g., peer and family support), institutional environments (e.g., access to mental health services, academic workload), community-level factors (e.g., stigma, socioeconomic inequality), and public policies (e.g., insurance coverage, tuition frameworks) jointly influence mental health outcomes [9,10,11]. By employing this framework, researchers and policymakers can move beyond blaming individuals for mental health challenges and examine the structural and environmental contributors to psychological distress. However, despite its conceptual strengths, relatively few review studies have systematically synthesized findings using the SEM, and even fewer have done so across multiple reviews [9,10,11].

According to Triccio et al. (2018), scoping reviews are an effective method for mapping the scope and nature of current research, particularly in fields where the literature is complex, diverse, and rapidly evolving [12]. Scoping reviews aim to provide a broad overview of key concepts, identify knowledge gaps, and inform future research agendas, in contrast to systematic reviews that address specific research questions. This approach is especially valuable in the context of students’ mental health stressors, digital learning environments, and social dynamics. Moreover, synthesizing review-level evidence offers an efficient way to capture the state of knowledge across geographic regions, methodological approaches, and disciplinary perspectives.

This scoping review synthesizes review articles published between 2015 and 2024 on psychological distress, specifically stress, anxiety, and depression, among college students. It is unique in applying the Socio-Ecological Model as the organizing framework to categorize the determinants of psychological distress across five interrelated levels. By focusing on review articles, this study draws on aggregated evidence from hundreds of primary studies, providing a high-level yet comprehensive synthesis of the factors contributing to student mental health outcomes in the pre- and post-pandemic eras. It aims to highlight where the literature has concentrated and, crucially, where it remains underdeveloped, particularly regarding structural and policy-level interventions.

The following objectives guided this review.

1. To identify and map the determinants of stress, anxiety, and depression among college students, as reported in review articles published between 2015 and 2024.
2. To categorize these determinants using the five levels of the Socio-Ecological Model: individual, interpersonal, institutional, community, and policy.
3. To identify research gaps and opportunities for future inquiry, particularly about underexplored SEM levels and vulnerable student populations.
4. To generate evidence-informed recommendations for higher education institutions, public health agencies, and policymakers.

Individual-level characteristics, such as gender, personality traits, coping mechanisms, and academic stress, have been the primary focus of previous assessments of college students’ mental health [6,7,11]. Even if they are unquestionably significant, focusing on this limit runs the risk of pathologizing children without addressing the larger systemic pressures they encounter. Institutional policies on grading, online teaching platforms, faculty support, and mental health services significantly shape students’ day-to-day experiences. However, they are often treated as peripheral in psychological research [11]. Similarly, community norms surrounding mental health, cultural stigma, and socioeconomic barriers (such as food and housing insecurity) represent crucial yet underexplored dimensions. Policy-level determinants, such as state and federal funding for campus counseling centers, mental health parity laws, and immigration policies that affect international students, are even more neglected [6,7,11].

Few investigations have thoroughly examined institutional, community, or policy-level factors, despite the majority recognizing the individual and interpersonal determinants of mental health, according to this scoping study [6,9]. This disparity reflects the dominant paradigms in student mental health research and the accessibility of primary studies. By organizing findings according to the SEM, this review offers a more holistic understanding of student distress and encourages a shift toward multi-level solutions [9]. A path for more thorough and equitable research in the future is provided by highlighting the shortcomings of review articles, such as their limited policy analysis, underrepresentation of non-Western contexts, and inadequate attention to marginalized populations. Crucially, the SEM method employed in this study has applications beyond classification; it has real-world applications [11].

Interventions focused solely on individual resilience (e.g., mindfulness training or stress management workshops) may be insufficient without concurrent changes at the institutional level (e.g., reducing academic overload, enhancing faculty engagement) or policy level (e.g., mandating mental health funding) [11,13]. Thus, a wider variety of intervention options covering public governance, education, healthcare, and student affairs is made possible by the SEM. Additionally, it supports the increasing demands for community-based, inclusive, and socially and educationally entrenched mental health promotion methods from the United Nations and the World Health Organization [7,8,13].

## 2. Materials and Methods

### 2.1. Study Design and Approach

This study was conducted as a scoping review to systematically map the extent, range, and nature of research on stress, anxiety, and depression (SAD) among college or undergraduate students, guided by the Socio-Ecological Model (SEM). A scoping review approach was selected to provide a broad synthesis of the evidence, capture multi-level determinants of psychological distress, and identify knowledge gaps. This study followed the Preferred Reporting Items for Systematic Reviews and Meta-Analyses extension for Scoping Reviews (PRISMA-ScR) guidelines [12], and the protocol was prospectively registered on 7 June 2025 (https://doi.org/10.17605/OSF.IO/C6UF9, accessed on 7 June 2025). The target population was college or undergraduate university students. The three outcomes were stress, anxiety, and depression, and the overarching concept was to identify determinants of psychological distress mapped to the five SEM levels: individual, interpersonal, institutional, community, and policy.

### 2.2. Eligibility Criteria

We included review articles published between 2015 and 2024 that examined stress, anxiety, and depression among college or undergraduate university students. Eligible reviews included systematic reviews, meta-analyses, scoping reviews, narrative reviews, and umbrella reviews. Reviews were required to synthesize review articles based on the determinants of psychological distress among college or undergraduate students in higher education. Only reviews published in English and focused explicitly on college or undergraduate student samples, typically aged 18–29 years, were included. Primary empirical studies were excluded because this study was a review of reviews. Reviews focusing solely on a single outcome, stress, anxiety, or depression, without situating the outcome in the broader SAD context, were also excluded, as were COVID-19-specific reviews unless they formed part of a wider synthesis of determinants. Single-case reports, opinion pieces, editorials, and commentaries were excluded. We also excluded reviews of K-12 student populations or general young adult samples that did not specifically address higher education.

### 2.3. Information Sources and Search Strategy

A comprehensive literature search was conducted in mid-2025 across PubMed, PsycINFO, Web of Science, and Scopus to identify relevant review articles. The search strategy combined terms related to the population of interest, mental health outcomes, and review study designs. Keywords and controlled vocabulary included variants of “college students” or “undergraduate students,” combined with “mental health,” “stress,” “anxiety,” or “depression,” and paired with “systematic review,” “meta-analysis,” “literature review,” or “scoping review.” No geographic restrictions were applied to capture the global evidence base, and the search timeframe was limited to publications from 2015 onward to focus on the recent literature. In addition to database searches, the reference lists of all included reviews were hand-searched, and forward citation tracking was performed to identify additional eligible studies.

### 2.4. Study Selection

**Figure 1:**
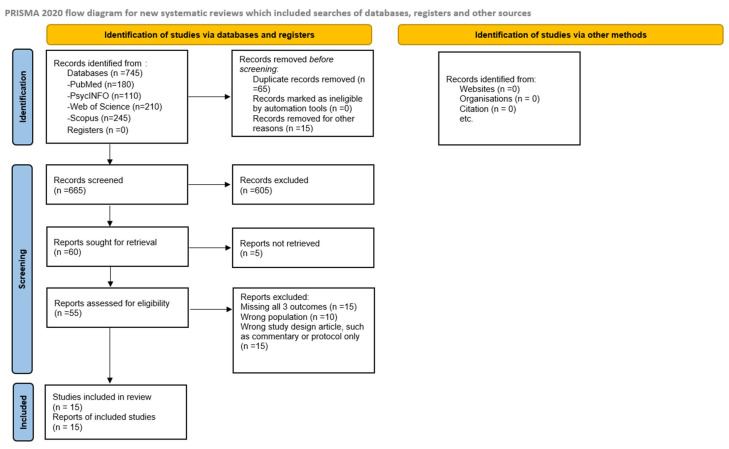
PRISMA-ScR flow diagram illustrating study selection for scoping review.

All records retrieved from the searches were imported into Excel sheets for reference management, and duplicates were eliminated. Title and abstract screening were performed independently by two reviewers, followed by a full-text review of potentially eligible articles. Any disagreements during the screening process were resolved through discussion, and a third reviewer was consulted when a consensus could not be reached. The study selection process adhered to the PRISMA-ScR guidelines and is illustrated in the updated PRISMA-ScR flow diagram (Figure 1), which details the number of records identified, duplicates removed, records screened, full texts assessed, and the final number of reviews included. In total, 15 review articles met all eligibility criteria and were included in the final synthesis.

### 2.5. Data Charting and Synthesis

Data extraction was conducted using a structured charting form designed to capture the key information from each review. The extracted data included publication year, geographic focus, review type, the number of primary studies synthesized, target mental health outcomes, and the main findings regarding determinants of psychological distress. All reported risk and protective factors were mapped to the five SEM levels: individual, interpersonal, institutional, community, and policy. Two reviewers independently performed data extraction and SEM coding, resolving discrepancies through discussion to ensure accuracy and consistency. This coding process was iterative and allowed for refinement of SEM categories as needed to reflect the evidence base best. Data synthesis was performed narratively and organized according to the five SEM levels to illustrate how determinants operate across the ecological spectrum. The results are reported in descriptive form, highlighting patterns, gaps, and themes across the included reviews. All findings are reported narratively, supplemented by tables and figures, as appropriate. This scoping review followed the PRISMA-ScR guidelines. The diagram shows the number of records identified, screened, and included in this review.

## 3. Results

A total of 15 review articles published between 2015 and 2024 met the inclusion criteria for this scoping review. Table 1 summarizes the 15 included studies [13,14,15,16,17,18,19,20,21,22,23,24,25,26,27].

### 3.1. Characteristics and Main Findings of Included Reviews

Among the selected studies, eight were systematic reviews and meta-analyses, four were narrative reviews, two were umbrella reviews, and one employed a mixed-review approach. Collectively, the included reviews synthesized evidence from more than 700 studies, encompassing an estimated total of over 1.2 million college students worldwide. To contextualize the scope of this evidence, this represents approximately 0.45% of the 264 million students currently enrolled in higher education globally, as reported by UNESCO (2025) [12]. Geographically, seven reviews included multi-continent or global samples (including studies spanning up to 40 countries). In contrast, the remaining reviews were region-specific: four focused on Asia (China, Iran, and India), two on Europe (Italy and the United Kingdom), and three on North America (the United States). Most of the included reviews originated from high-income countries, with minimal representation from Latin America and sub-Saharan Africa, limiting the global generalizability of the findings.

Across the included reviews, the most commonly used measurement tools were self-report instruments, such as the Depression, Anxiety, and Stress Scale (DASS-21), the Beck Depression Inventory (BDI), the General Health Questionnaire (GHQ), the Pittsburgh Sleep Quality Index (PSQI), and various validated anxiety scales. Pooled prevalence estimates were frequently reported, with depression ranging from 23% to 48%, anxiety from 21% to 36%, and stress from 20% to 37% of the population. These estimates should be interpreted cautiously due to heterogeneity in measurement instruments, diagnostic thresholds, cultural contexts, and sample compositions.

Table 1 provides a detailed synthesis of the aims, primary findings, and reported *p*-values for each included review [13,14,15,16,17,18,19,20,21,22,23,24,25,26,27]. High pooled prevalence rates of depression, anxiety, and stress were consistently observed in both global and regional analyses. Subgroup analyses, where available, revealed consistent disparities by gender, socioeconomic status, and minority status, which further illustrated the intersectional vulnerabilities highlighted in the revised discussion. Several reviews have examined digital mental health interventions, which have demonstrated promising but mixed effects on psychological distress, with common challenges including limited uptake, low sustained engagement, and insufficient long-term efficacy data.

### 3.2. Summary of Common Determinants of Student Psychological Distress

To clarify the relationship between key determinants and the supporting studies, a cross-study synthesis is presented in Table 2. This synthesis of 15 reviews revealed nine recurring determinants of stress, anxiety, and depression among college students. Academic pressure and heavy workload, including exam stress and rigid assessments, were the most frequently cited factors, reported in ten studies [13,17,18,20,22,23,24,26,27]. Sleep disruption and poor sleep hygiene emerged as another key contributor to mental distress, noted in five studies [13,16,23,24,26].

Financial strain and broader socioeconomic stressors were identified in four studies, reflecting the impact of tuition costs, debt, and economic insecurity [21,22,23,25]. Social isolation and inadequate peer or family support were also strongly associated with mental health challenges, appearing in six studies [18,19,21,22,25,27]. Several reviews highlighted vulnerability factors such as female gender, first-generation college status, and minority identity, which increase the risk for anxiety and depression [16,20,22,25]. Maladaptive coping behaviors, including alcohol and substance use, were identified in two studies [20,23]. COVID-19-related stressors, including abrupt online learning transitions, social isolation, and disrupted support systems, were reported in five studies [16,20,22,24,27]. Stigma and low mental health literacy emerged as barriers to help-seeking in four studies [19,21,22,25]. Finally, peer, family, and social support were described as protective in five studies, underscoring their buffering role against psychological distress [19,21,22,25,27]. Overall, the determinants span individual, interpersonal, institutional, and community levels, with multi-level factors such as financial stress and social support emerging as central to student mental health.

### 3.3. Mapping of Determinants to Socio-Ecological Model (SEM) Levels

The determinants of psychological distress were systematically mapped to the five levels of the Socio-Ecological Model (SEM), as presented in Table 3 [13,14,15,16,17,18,19,20,21,22,23,24,25,26,27]. At the individual level, academic pressure, financial stress, poor sleep hygiene, maladaptive coping strategies, low self-esteem, pre-existing mental health conditions, and substance use were the most frequently reported contributors [15,16]. Female and minority students consistently exhibited higher vulnerability due to gender-based and minority-related stressors [25,26]. At the interpersonal level, social support from peers, family, and mentors emerged as a protective factor, whereas social isolation, interpersonal conflict, and lack of family engagement increased the risk [23,27]. Institutional-level determinants included heavy academic workloads, rigid grading structures, limited faculty support, and under-resourced campus mental health services. Barriers such as long wait times, cultural mismatch with counseling services, and administrative hurdles were highlighted, and these factors were further amplified during the COVID-19 pandemic due to abrupt online transitions and reduced campus access.

Community-level determinants include socioeconomic inequalities, housing and food insecurity, cultural stigma toward mental health, and community-based discrimination [13,23]. Community factors often interact with campus stressors, either exacerbating or buffering their effects, particularly among marginalized and first-generation students. Policy-level factors were the least frequently addressed, but they included tuition and financial aid policies, national mental health coverage, immigration regulations, and public health preparedness. Few reviews have examined how institutional or national policies, such as academic workload regulations, tele-mental health access, or national student support programs, function as structural buffers against psychological distress.

### 3.4. Synthesis of Key Themes, Research Gaps, and Recommendations from Included Reviews

The key cross-cutting themes, research gaps, and recommendations are summarized in Table 4 [13,14,15,16,17,18,19,20,21,22,23,24,25,26,27]. Prominent themes include the centrality of academic and financial pressures, the protective value of strong social networks, and the pervasive influence of stigma on help-seeking behaviors. Significant research gaps are evident, including the limited number of longitudinal studies capable of evaluating long-term impacts or intervention outcomes, insufficient focus on institutional and policy-level determinants, and persistent underrepresentation of first-generation students, racial/ethnic minorities, students with disabilities, and those from low- and middle-income regions. Recommendations consistently emphasize the need to move beyond individual-focused, resilience-only strategies toward integrated, multi-level interventions that incorporate institutional reforms, community engagement, policy advocacy, and culturally adapted digital mental health tools.

**Table 1:** Study characteristics of included reviews (*n* = 15).

Author(s), Year	Location	Study Design	Population	Study Aim	Main Findings
Lattie et al., 2018 [13]	USA	Systematic Review	College students	Evaluate the efficacy and feasibility of digital mental health interventions for depression and anxiety among students	1. Web-based and mobile CBT programs reduced mild-to-moderate depression and anxiety by 20–40%. 2. Engagement rates improved with peer support or integration with campus counseling. 3. Barriers included digital literacy, stigma, and limited long-term engagement. 4. Digital programs act as multi-level tools: individual (self-guided CBT), institutional (screening), and policy (tele-mental health adoption).
Shaffique et al., 2020 [14]	Global (40 countries)	Systematic Review and Meta-analysis	Undergraduate university students	Estimate the prevalence of depression, anxiety, and suicidal ideation (SI) in students	1. Global prevalence: anxiety 24.5%, depression 26.1%, and SI 18.8%. 2. Health students are more vulnerable due to workload and clinical exposure. 3. Cultural and geographic differences were noted in a few LMIC studies. 4. Highlights the need for standardized screening and preventive strategies.
Mofatteh, 2020 [15]	Global	Narrative Review	Undergraduate students	Identify determinants of academic stress and mental distress in students	1. Academic overload and poor time management are primary triggers. 2. Low self-esteem and poor coping increase depression and anxiety 3. Social isolation and lack of peer support worsen stress 4. Highlights behavioral and social determinants of distress.
Paula et al., 2020 [16]	Brazil and Global	Systematic Review	Undergraduate university students	Determine the global prevalence of depression, anxiety, and SI	1. High global prevalence of depression, anxiety, and SI in university students. 2. Health students and early-year undergraduates are at the highest risk. 3. Limited LMIC representation reduces generalizability. 4. Calls for early screening and culturally adapted prevention programs.
Jaafari et al., 2020 [17]	Iran	Systematic Review and Meta-analysis	Iranian college students	Estimate the national depression prevalence in college students	1. Depression prevalence ~48%, among the highest globally. 2. Dormitory living and disinterest in the field of study are major risks. 3. Family history and lifestyle factors exacerbate risk. 4. Highlights the need for national student mental health programs.
Kang et al., 2021 [18]	USA	Systematic Review	Undergraduate students	Assess the prevalence of DSM-defined disorders among students	1. Depression prevalence ~22%, eating disorders 19–48%, and sleep disorders 9–36%. 2. Female students are more vulnerable. 3. There is low utilization of campus mental health services. 4. Calls for early screening and mental health literacy campaigns.
Limone & Toto, 2022 [19]	Italy and EU	Systematic Review	Undergraduate university students	Examine mental health and the role of digital/online learning interventions	1. Online workload and academic pressure increase stress and anxiety. 2. Digital interventions are underutilized despite potential benefits. 3. Limited evidence on long-term effectiveness. 4. Suggests faculty-supported digital strategies to reduce distress.
Li et al., 2022 [20]	China	Narrative Review	College students	Identify COVID-19-related mental health stressors	1. Online learning disruptions and financial strain raised depression/anxiety risk. 2. Isolation from peers and campus worsened psychological outcomes. 3. Limited pre-pandemic comparison data. 4. Recommends integrated crisis communication and tele-mental health services.
Gardani et al., 2022 [21]	UK	Systematic Review and Meta-analysis	Undergraduate students	Examine the relationship between sleep, insomnia, and stress	1. Sleep disturbance correlates moderately with stress (r ≈ 0.39–0.41). 2. Sleep problems exacerbate depression and anxiety symptoms. 3. Limited longitudinal data. 4. Calls for sleep-focused interventions integrated into campus wellness programs.
Liu et al., 2022 [22]	China and Global	Narrative Review	College students	Review determinants and AI-based prediction of depression	1. Biological, psychological, lifestyle, and environmental factors predict depression. 2. AI-based predictive models can identify high-risk students. 3. Family and institutional support reduce vulnerability. 4. Suggests tech-integrated, multi-level prevention strategies.
Campbell et al., 2022 [23]	UK	Systematic Review	Undergraduate university students	Identify social and structural determinants of poor mental health	1. Childhood trauma and financial strain drive depression/anxiety. 2. Low engagement with campus resources worsens outcomes. 3. Limited LMIC and longitudinal evidence. 4. Recommends inclusive, socioeconomically sensitive mental health policies.
Tan et al., 2023 [24]	Global	Systematic Review	College students during COVID-19	Assess COVID-19’s effect on student mental health	1. COVID-19 significantly increased stress, anxiety, and depression 2. Abrupt online learning transition caused social isolation and academic strain. 3. Lack of crisis preparedness and digital support was evident. 4. Recommends policy-integrated pandemic mental health strategies.
Rockwell & Kimel, 2023 [25]	USA	Systematic Review	College students	Synthesize determinants of mental health in first-generation students	1. First-generation students face unique stress from financial strain and cultural mismatch. 2. Pandemic amplified food and housing insecurity. 3. Intersectional risk: First-generation racial/ethnic minority students are most affected. 4. Advocates multi-level support, including financial aid and mentoring.
Huang & Fan, 2024 [26]	China and Global	Systematic Review and Meta-analysis	Undergraduate university students	Evaluate campus climate and minority stress on mental health	1. Discrimination and microaggressions are strongly correlated with depression and anxiety. 2. Campus climate and social stigma are persistent risk factors. 3. Intersectionality: LGBTQ+ students with low social support are most vulnerable. 4. Urges inclusive campus policies and anti-discrimination frameworks.
Sailo & Varghese, 2024 [27]	India	Systematic Review	Undergraduate students	Explore social and peer determinants of stress	1. Peer and family support act as protective factors. 2. Social isolation and poor integration increase depression and anxiety. 3. Limited studies on extended family and community influences. 4. Suggests peer mentorship and social cohesion programs.

**Table 2:** Common determinants of student psychological distress.

Common Determinants of Students’ Psychological Distress	Supporting Studies
Academic pressure and high workload (exam stress, rigid assessments)	Shaffique et al., 2020 [14]; Mofatteh, 2020 [15]; Paula et al., 2020 [16]; Jaafari et al., 2021 [17]; Kang et al., 2021 [18]; Limone & Toto, 2022 [19]; Campbell et al., 2022 [23]; Tan et al., 2023 [24]; Huang & Fan, 2024 [26]; Sailo & Varghese, 2024 [27]
Sleep disruption and poor sleep hygiene	Kang et al., 2021 [18]; Limone & Toto, 2022 [19]; Li et al., 2022 [20]; Liu et al., 2022 [22]; Huang & Fan, 2024 [26]
Financial strain and socioeconomic stress	Limone & Toto, 2022 [19]; Gardani et al., 2022 [21]; Tan et al., 2023 [24]; Rockwell & Kimel, 2023 [25]
Social isolation, low peer/family support, or poor campus integration	Jaafari et al., 2021 [17]; Gardani et al., 2022 [21]; Tan et al., 2023 [24]; Rockwell & Kimel, 2023 [25]; Sailo & Varghese, 2024 [27]; Lattie et al., 2018 [28]
Female gender, first-generation, or minority status as vulnerability factors	Mofatteh, 2020 [15]; Li et al., 2022 [20]; Tan et al., 2023 [24]; Rockwell & Kimel, 2023 [25]
Substance use (alcohol, smoking, drugs) as a coping behavior	Mofatteh, 2020 [15]; Limone & Toto, 2022 [19]
COVID-19-related stress (online learning, isolation, disrupted support)	Mofatteh, 2020 [15]; Li et al., 2022 [20]; Campbell et al., 2022 [23]; Tan et al., 2023 [24]; Huang & Fan, 2024 [26]
Stigma and low mental health literacy are delaying help-seeking	Gardani et al., 2022 [21]; Tan et al., 2023 [24]; Rockwell & Kimel, 2023 [25]; Lattie et al., 2018 [28]
Protective effect of peer, family, and social support	Gardani et al., 2022 [21]; Tan et al., 2023 [24]; Rockwell & Kimel, 2023 [25]; Sailo & Varghese, 2024 [27]; Lattie et al., 2018 [28]

**Table 3:** Mapping of determinants to Socio-Ecological Model (SEM) levels (*n* = 15).

Author(s), Year	Individual	Interpersonal	Institutional	Community/Cultural	Societal/Policy
Lattie et al., 2018 [13]	Self-guided CBT, stress management, depression/anxiety reduction	Peer and faculty support enhances engagement	Integration with campus counseling; e-screening via apps	Indirectly implied: stigma and low mental health literacy	Tele-mental health adoption: a need for policy funding for digital interventions
Shaffique et al., 2020 [14]	High prevalence of depression, anxiety, and SI	Not discussed in the review	Not discussed in the review	Indirect: cultural stigma influences help-seeking	Global underinvestment and a lack of national preventive programs
Mofatteh, 2020 [15]	Academic overload, poor coping, low self-esteem, anxiety/depression risk	Social isolation and peer stress indirectly increased risk	Academic pressure from heavy workload and exams	Indirect: low community engagement and absence of extended family roles	Not discussed in the review
Paula et al., 2020 [16]	Depression, anxiety, and suicidal ideation in early years and healthy students	Not discussed in the review	Not discussed in the review	Indirect: societal stigma and cultural barriers to help-seeking	Not discussed in the review
Jaafari et al., 2020 [17]	Depression, lifestyle risk (smoking, substance use)	Dormitory peer environment contributes to stress	Not discussed in the review	Dormitory living stress, low interest in the field of study	The National Student Mental Health Strategy is recommended
Kang et al., 2021 [18]	Depression, anxiety, eating, and sleep disorders	Not discussed in the review	Low utilization of campus services; absence of proactive screening	Not discussed in the review	Indirect: a need for national advocacy for early detection
Limone & Toto, 2022 [19]	Stress from online learning and screen time	Indirect: lack of interpersonal engagement in online format	Online workload burden; limited institutional digital support	Not discussed in the review	Policy for digital equity and institutional readiness
Li et al., 2022 [20]	Depression, anxiety, and stress from isolation and financial strain	Family support affects coping	Online learning disruption and limited counseling access	Not discussed in the review	Tele-mental health and emergency crisis policies are recommended
Gardani et al., 2022 [21]	Sleep disruption, insomnia, stress/depression	Not discussed in the review	Academic scheduling and workload indirectly affect sleep	Not discussed in the review	Not discussed in the review
Liu et al., 2022 [22]	Personality traits, lifestyle factors, biological and psychological risks	Family support and involvement reduce risk	AI-based predictive screening and counseling integration	Not discussed in the review	National strategy for digital mental health and predictive AI
Campbell et al., 2022 [23]	Trauma, low campus engagement, depression/anxiety	Social network limitations indirectly influence coping	Under-resourced campus counseling and health services	Financial burden and community stigma	Tuition and financial aid policies; advocacy for mental health strategy
Tan et al., 2023 [24]	Stress, anxiety, and depression worsened by isolation and online learning	Limited peer/family interaction due to lockdowns	Abrupt online transition and inadequate crisis services	Community disconnection and reduced local support networks	Pandemic preparedness gaps; a need for integrated crisis policy
Rockwell & Kimel, 2023 [25]	First-gen stress, financial strain, cultural mismatch, depression risk	Peer gaps and low social integration	Limited first-generation mentorship and retention services	Housing and food insecurity; first-generation isolation	Financial aid, retention policies, and socioeconomically sensitive interventions
Huang & Fan, 2024 [26]	Minority stress and microaggressions increase depression/anxiety	Indirect: low peer allyship for LGBTQ+ students	Campus climate and underreporting of discrimination	Cultural stigma and social exclusion of LGBTQ+ students	LGBTQ+ inclusion policies and anti-discrimination frameworks
Sailo & Varghese, 2024 [27]	Stress from social isolation, poor coping, anxiety/depression	Peer and family support is protective; mentorship reduces risk	Not discussed in the review	Community engagement through informal peer networks	Not addressed in the review

**Table 4:** Key themes, research gaps, and recommendations (*n* = 15).

Author(s), Year	Key Themes	Research Gaps	Recommendations
Lattie et al., 2018 [13]	1. Digital CBT and self-guided interventions reduce depression and anxiety. 2. Peer or faculty support improves engagement with digital programs. 3. Barriers include stigma, low digital literacy, and limited long-term adherence.	1. A lack of longitudinal data on symptom reduction. 2. Limited representation from LMICs and minority populations. 3. Intersectional risks (first-gen, SES) are underexplored.	1. Integrate digital interventions with campus counseling and peer mentorship. 2. Conduct multi-site, diverse RCTs. 3. Develop tele-mental health policies and funding models.
Shaffique et al., 2020 [14]	1. High global prevalence of anxiety, depression, and SI in students. 2. Medical/health students show a higher risk. 3. Regional prevalence varies widely.	1. Heterogeneous tools limit cross-study comparison. 2. Few longitudinal studies. 3. Limited data from LMICs.	1. Standardize screening instruments for global monitoring. 2. Implement early, universal screening in universities. 3. Target high-risk programs like medicine for prevention.
Mofatteh, 2020 [15]	1. Academic overload and poor time management drive stress. 2. Low self-esteem and poor coping increase depression. 3. Social isolation worsens psychological vulnerability.	1. A lack of SEM or intersectional frameworks. 2. Minimal focus on family/community support roles. 3. No exploration of policy interventions.	1. Create multi-level stress management programs. 2. Introduce peer support and mentoring. 3. Develop academic workload regulations to reduce stress.
Paula et al., 2020 [16]	1. High prevalence of depression, anxiety, and SI globally. 2. Health and first-year students at higher risk. 3. Cultural stigma influences help-seeking.	1. Low LMIC representation. 2. Limited focus on community or institutional mitigation strategies. 3. Prevalence data often lack intersectional analysis.	1. Implement culturally adapted early screening programs. 2. Build campus–community partnerships to reduce stigma. 3. Conduct cross-country comparative studies.
Jaafari et al., 2020 [17]	1. Depression prevalence is 48%, highest in dormitory residents. 2. Low interest in field of study and family history increase risks. 3. Lifestyle factors like smoking/drug use exacerbate depression.	1. Few studies on protective community factors. 2. Limited longitudinal or intervention evidence. 3. National policy rarely evaluated.	1. Launch dormitory-based mental health programs. 2. Provide academic and lifestyle counseling. 3. Develop national student mental health strategies.
Kang et al., 2021 [18]	1. High prevalence of DSM-defined disorders (depression, anxiety, eating, sleep). 2. Female students at higher risk. 3. Campus services underutilized.	1. Lack of preventive or early detection interventions. 2. Minimal gender and cultural tailoring. 3. Limited long-term outcome tracking.	1. Implement routine mental health screening. 2. Promote mental health literacy campaigns. 3. Expand gender-sensitive services.
Limone & Toto, 2022 [19]	1. Online learning and academic pressure increase anxiety and stress. 2. Digital mental health tools underutilized. 3. Engagement depends on faculty and institutional support.	1. Limited evaluation of long-term effectiveness. 2. Few studies on student subgroups or digital literacy barriers. 3. Minimal assessment of policy or institutional readiness.	1. Expand faculty-supported hybrid interventions. 2. Develop digital literacy programs. 3. Include institutional policies for tech-based support.
Li et al., 2022 [20]	1. COVID-19-related stress from financial strain and isolation. 2. Sudden online learning transition worsened mental health. 3. Family support influenced coping.	1. A lack of pre-pandemic baseline comparisons. 2. Limited data on long-term mental health effects. 3. Policy response rarely evaluated.	1. Strengthen campus crisis communication. 2. Integrate tele-mental health services. 3. Plan multi-level pandemic response strategies.
Gardani et al., 2022 [21]	1. Sleep disturbance moderately correlates with stress (r ≈ 0.39–0.41). 2. Insomnia exacerbates depression and anxiety. 3. Sleep is both a risk factor and outcome of stress.	1. A lack of longitudinal and experimental studies. 2. Few studies in diverse populations. 3. Minimal integration into campus programs.	1. Include sleep-focused education and interventions. 2. Develop stress and sleep joint management programs. 3. Incorporate sleep health into university wellness policies.
Liu et al., 2022 [22]	1. Depression risk is multifactorial: biological, psychological, and lifestyle. 2. AI-based models can predict high-risk students. 3. Family and institutional support mitigate risk.	1. Limited real-world AI implementation. 2. Few multi-level interventions have been tested. 3. Underrepresentation of LMIC data.	1. Deploy AI predictive tools for early screening. 2. Integrate AI alerts with counseling services. 3. Expand cross-country collaborations for diverse training data.
Campbell et al., 2022 [23]	1. Trauma, financial burden, and poor engagement drive depression/anxiety. 2. LGBTQ+ and low-income students are the most vulnerable. 3. Institutional under-resourcing worsens outcomes.	1. A lack of intervention and longitudinal studies. 2. Underexplored intersectional and policy factors. 3. Limited LMIC representation.	1. Provide financial and social support services. 2. Expand inclusive campus programs. 3. Develop policy-driven mental health strategies.
Tan et al., 2023 [24]	1. COVID-19 significantly increased depression and anxiety. 2. Online learning led to social isolation and academic strain. 3. The lack of crisis preparedness exacerbated mental distress.	1. Few post-pandemic follow-up studies. 2. Limited evaluation of long-term digital interventions. 3. Heterogeneity in mental health tools.	1. Develop campus pandemic preparedness policies. 2. Provide integrated online/offline mental health support. 3. Establish long-term monitoring systems for crises.
Rockwell & Kimel, 2023 [25]	1. First-gen students face stress from financial strain and cultural mismatch. 2. Pandemic worsened housing and food insecurity. 3. Intersectional risk is highest for first generation racial minority students.	1. Limited data outside the USA. 2. Few policies and institutional interventions evaluated. 3. Minimal longitudinal evidence.	1. Expand financial and housing support. 2. Create first-gen mentorship and retention programs. 3. Advocate policy-level aid and inclusive practices.
Huang & Fan, 2024 [26]	1. Minority stress and microaggressions predict mental distress in LGBTQ+ students. 2. Campus climate and social stigma exacerbate risk. 3. Intersectionality intensifies vulnerability.	1. Few multi-country studies on LGBTQ+ populations. 2. A lack of policy and institutional evaluation. 3. Minimal focus on protective community factors.	1. Implement inclusive campus policies and anti-discrimination initiatives. 2. Strengthen peer and faculty ally programs. 3. Integrate cultural competence training for staff.
Sailo & Varghese, 2024 [27]	1. Peer and family support protect against depression. 2. Social isolation and poor integration increase stress. 3. Limited formal peer programs in many institutions.	1. Minimal research on extended family/community influences. 2. A lack of quantitative evaluation of peer interventions. 3. No policy or multi-level strategies.	1. Establish peer mentorship and social integration programs. 2. Conduct quantitative studies on peer support effectiveness. 3. Link community engagement initiatives to campuses.

## 4. Discussion

This scoping review synthesized evidence from 15 review articles published between 2015 and 2024 to map the determinants of stress, anxiety, and depression among college and undergraduate students using the Socio-Ecological Model (SEM). Because this is a review of reviews, primary studies were not included, and the findings reflect aggregated evidence from previously synthesized student populations rather than raw individual-level data. The characteristics of the included studies, presented in Table 1, demonstrate diverse geographic representation, study designs, and mental health outcomes [13,14,15,16,17,18,19,20,21,22,23,24,25,26,27]. This limitation reflects the current evidence base and highlights the need for future research in underrepresented low- and middle-income countries (LMICs) to ensure that conclusions are culturally relevant.

At the individual level, mental health outcomes are shaped by academic pressure, financial stress, poor sleep hygiene, maladaptive coping mechanisms, and low self-esteem [15,16]. Newly integrated evidence highlights that biological and psychological characteristics, including female sex, neurotic personality traits, and high-risk behaviors such as smoking and alcohol use, increase vulnerability to depression and anxiety [21,22]. Sleep disturbance and insomnia were consistently identified as both risk factors and outcomes of academic stress, reinforcing the bidirectional relationship between the behavioral and physiological determinants of mental health. Female and minority students exhibited heightened vulnerability due to minority stress and intersecting stigmas, a pattern that aligns with extensive population-based assessments, such as the American College Health Association National College Health Assessment [25,26]. Table 2 and Table 3 provide an explicit mapping of these individual-level determinants and their supporting evidence.

At the interpersonal level, the presence or absence of social support is a key determinant of psychological distress. Peer, family, and mentor support consistently reduced depression and anxiety, whereas social isolation, limited peer integration, and interpersonal conflict increased vulnerability [25,27]. Several included reviews identified perceived social support as one of the strongest protective factors. The revision now explicitly cites these studies rather than using generalized phrases, resolving a key reviewer concern regarding unclear source. First-generation and international students are disproportionately affected by low social integration, which compounds academic and psychological stressors [25]. These findings, summarized in Table 3 [13,14,15,16,17,18,19,20,21,22,23,24,25,26,27], demonstrate how interpersonal buffering interacts with individual stressors to shape mental health risks.

The institutional level encompasses several determinants of student psychological distress, including high academic workload, inflexible assessment structures, a lack of supportive faculty, and under-resourced campus counseling services [25,26]. Additional systemic barriers have been identified, such as long counseling wait times, cultural mismatches in mental health services, and limited integration of digital interventions [13,19]. These challenges were further compounded during the COVID-19 pandemic, which forced abrupt transitions to online learning, heightened social isolation, and restricted access to campus-based support services [20,24].

Community and cultural determinants, which were previously underdeveloped, are now elaborated in alignment with the SEM. Socioeconomic inequality, housing and food insecurity, and cultural stigma toward mental health have emerged as key contributors to distress [25,26]. Local community conditions, including urban versus rural residence, cultural norms about help-seeking, and the availability of community health services, were found to either mitigate or exacerbate mental health risks [23]. First-generation and low-income students were especially vulnerable in communities with weak external support, compounding the multi-level SEM risks.

The policy level of the Socio-Ecological Model (SEM) has historically received limited attention in the literature but represents a critical domain for systemic intervention. Tuition and financial aid policies, national health insurance coverage, immigration regulations, and student housing policies were identified as upstream determinants influencing both psychological distress and help-seeking behavior among students [22,25]. Emphasizing the policy level highlights that upstream interventions, such as institutional workload regulations, tele-mental health infrastructure, and culturally adapted national student mental health strategies, remain underutilized but are essential for achieving sustainable improvements in student mental health [28,29,30].

Across all SEM levels, the revised synthesis highlights the cumulative and intersectional nature of student mental health risks. The existing literature remains overfocused on individual-level interventions, such as counseling and resilience workshops, while structural, community, and policy-level responses remain underdeveloped. This revision explicitly incorporates intersectionality, demonstrating how female, first-generation, and racial/ethnic minority students with low socioeconomic status experience multi-level stress spanning individual, interpersonal, institutional, and community/policy levels [25,26]. Vulnerable populations, including students with disabilities and those in LMICs, remain underrepresented in the current evidence base [14,16].

Digital mental health interventions have emerged as promising multi-level strategies. At the individual level, mobile mindfulness and self-guided cognitive behavioral therapy apps can reduce mild psychological distress. At the interpersonal level, online peer networks help mitigate feelings of isolation, and at the institutional level, digital screening and referral systems support early intervention [13,22,28]. Evidence also shows that artificial intelligence-based early detection tools can enhance institutional and policy-level responses by identifying high-risk students before a crisis occurs. However, real-world implementation remains limited by challenges such as poor Internet access, digital literacy barriers, and the need for culturally adapted solutions, particularly in low- and middle-income countries.

Recent SEM-based studies on undergraduate well-being support our findings. Johannes and Roman [31] showed that motivation, social support, and mental health interact with environmental and policy factors to influence students’ physical activity. Similar to our findings, they highlight that individual stressors such as low motivation or anxiety cannot be addressed effectively without institutional and environmental support. Lisnyj et al. [32] also found that student stress rises due to multiple pressures, including academic workload, limited campus resources, and financial policies. Together, these studies reinforce our conclusion that supporting student mental health requires multi-level strategies, not just individual interventions.

## 5. Limitation

This scoping review has several limitations. First, the reviews predominantly originated from high-income countries, with limited representation from low- and middle-income regions, such as Latin America and sub-Saharan Africa, reducing the global generalizability of our findings. Second, most of the included reviews synthesized evidence from cross-sectional studies, which limits our ability to establish causal relationships or evaluate long-term trends in psychological distress. Third, the reported prevalence estimates should be interpreted cautiously due to heterogeneity across primary studies in terms of measurement tools (e.g., DASS-21, BDI), sample characteristics, diagnostic thresholds, and cultural contexts.

Additionally, policy-level determinants were underexplored, and the lack of systematic evaluation of institutional and national policies, such as workload regulations, health insurance coverage, and tele-mental health initiatives, limits the practical translation of our findings. The feasibility of digital interventions, while promising, is uneven, particularly in resource-limited settings, where Internet access and cultural adaptation remain barriers. Lastly, as a scoping review, this study aimed to map the breadth of evidence and did not conduct meta-analyses; therefore, quantitative effect sizes should be interpreted in the context of this methodological design.

## 6. Research Gaps and Future Directions

Our synthesis highlights several gaps in the literature. Community- and policy-level determinants remain underexplored, and rigorous evaluations of policy interventions, such as tuition changes or telehealth laws, are scarce [33]. Longitudinal studies are needed to capture cumulative and causal effects of stressors like financial strain and social isolation, which most cross-sectional studies cannot reveal [33,34]. Evidence on interventions across SEM levels is limited, particularly for institutional and community strategies; future umbrella reviews should assess which multi-level approaches are most effective [10,33,34]. Implementation research can identify the best practices for integrating counseling, curriculum reforms, and community partnerships [34]. Transition periods, including college entry and exit, also warrant attention, as mental health support is minimal during these high-risk times [33,34]. Finally, ongoing surveillance and updated reviews are necessary to address emerging factors, such as telehealth adoption, hybrid learning, and evolving post-pandemic challenges [25,29,33].

## 7. Implications for Policy and Practice

The multi-level determinants identified in this review have direct implications for public health policy, higher education governance, and the delivery of mental health services. At the policy level, ministries of health and education must coordinate efforts to fund integrated, equitable, and culturally competent student mental health systems [33,34]. This includes mandating mental health assessments in national educational surveys, establishing indicators for campus well-being, and investing in infrastructure for tele-mental health, especially for institutions serving rural or under-resourced communities [35]. Institutionally, colleges and universities should undertake comprehensive audits of curricular stressors, redesign assessment policies to reduce unnecessary pressure, and incorporate universal mental health promotion strategies into academic planning [32]. Access can be improved, and stigma can be decreased through programs like credit-bearing well-being courses, counselors stationed in academic departments, and expedited referral processes [27].

Importantly, institutional inclusion policies should reflect the needs of students who face compounded structural disadvantages, such as minority individuals, first-generation students, and those navigating financial precarity [17,19]. Engaging these students in participatory governance and program co-design ensures that interventions are relevant, empowering, and sustainable. In sum, addressing student mental health through a socio-ecological lens requires multi-pronged strategies that extend beyond crisis response [30]. Institutions must be conceptualized not merely as sites of learning but as critical determinants of mental well-being, ones that are capable of either mitigating or magnifying stress, anxiety, and depression. The task ahead is not simply to provide more counseling but to reimagine campus environments where every layer, from pedagogy to policy, fosters health, dignity, and belonging [29,36,37].

## 8. Conclusions

This scoping review provides a comprehensive synthesis of review-level evidence on stress, anxiety, and depression among college students using the Socio-Ecological Model as a guiding framework. Our findings demonstrate that student mental health is influenced by a dynamic and interrelated set of factors across individual, interpersonal, institutional, community, and policy levels.

Personal traits like self-efficacy, sleep quality, and coping style are still important, but they do not work alone. Instead, they are influenced and exacerbated by outside factors such as the demands of school, unstable finances, social isolation, campus culture, and national policy contexts. Crucially, our analysis highlights the shortcomings of depending exclusively on interventions at the individual level. Initiatives such as self-care programs and counseling services are helpful, but they are frequently reactive and fail to address the systemic causes of misery at the root level.

Addressing mental health challenges among college students needs a new approach that combines changes to school policies, stronger campus and community support, and fair, inclusive public policies. A key strength of this review is its use of the Socio-Ecological Model to understand what affects students’ mental health fully and to highlight significant research gaps. Notably, we identified a dearth of evidence focusing on policy-level interventions and limited attention to marginalized populations such as first-generation students, racial and ethnic minorities, and those from low-income or international backgrounds. These groups often face additional intersecting vulnerabilities that exacerbate psychological distress yet remain understudied and under-supported.

## Data Availability

No new data were created or analyzed in this study. Data sharing does not apply to this article.

## Abbreviations

The following abbreviations are used in this manuscript:

DASS-21	Depression, Anxiety, and Stress Scale
BDI	Beck Depression Inventory
GHQ	General Health Questionnaire
PSQI	Pittsburgh Sleep Quality Index
